# A Cerebral Basis for Visual Discomfort and Visual Stress

**DOI:** 10.3390/vision10020034

**Published:** 2026-06-11

**Authors:** Paul B. Hibbard, Peter Allen, Jordi M. Asher, Katherine Batey, Beverley Burke, Jason J. Braithwaite, Geoff G. Cole, Caelan Dow, Bruce J. W. Evans, Anna Franklin, Sarah M. Haigh, Hillevi Hemphälä, Ian Hosking, Andrew Keyes, Chan-su Lee, Ute Leonards, Cathy Manning, John Maule, Naomi Miller, Karen Monet, Louise O’Hare, Olivier Penacchio, Gordon T. Plant, Georgie Powell, Alice Price, Andrew J. Schofield, Miroslav Slouka, Petroc Sumner, Cleo Valentine, Thomas Wilcockson, Sanae Yoshimoto, Arnold J. Wilkins

**Affiliations:** 1 School of Psychology, University of Stirling, Stirling FK9 4LA, UK; jordi.asher@stir.ac.uk (J.M.A.); c.dow@student.reading.ac.uk (C.D.); 2 Vision and Hearing Sciences, Anglia Ruskin University, Cambridge CB1 1PT, UK; peter.allen@aru.ac.uk; 3 Vision Though Colour, Newcastle upon Tyne NE3 3PF, UK; katherine@keyeseyecare.co.uk (K.B.); andrew@keyeseyecare.co.uk (A.K.); 4 School of Psychological Sciences, Birkbeck, University of London, London WC1E 7HX, UK; beverley.burke@bbk.ac.uk; 5 Psychology Department, Lancaster University, Lancaster LA1 4YF, UK; j.j.braithwaite@lancaster.ac.uk; 6 Department of Psychology, University of Essex, Colchester CO4 3SQ, UK; ggcole@essex.ac.uk (G.G.C.); arnold@essex.ac.uk (A.J.W.); 7 Department of Optometry and Visual Science, City St George’s, University of London, London EC1V 0HB, UK; bruce.evans.1@citystgeorges.ac.uk; 8 Sussex Colour Group & Baby Lab, School of Psychology, University of Sussex, Brighton BN1 9RH, UK; anna.franklin@sussex.ac.uk; 9 Department of Psychology, University of Nevada, Reno, NV 89557, USA; shaigh@unr.edu; 10 Division of Ergonomics and Aerosol Technology, Institution of Design Sciences, Lund University, P.O. Box 118, 221 00 Lund, Sweden; hillevi.hemphala@design.lth.se; 11 Department of Engineering, University of Cambridge, Cambridge CB2 1PZ, UK; imh29@cam.ac.uk; 12 School of Electronic Engineering, Communication Engineering & Computer Science, Yeungnam University, Gyeongsan-si 38541, Gyeongsangbuk-do, Republic of Korea; chansu@ynu.ac.kr; 13 School of Psychology and Neuroscience, University of Bristol, Bristol BS8 1TU, UK; ute.leonards@bristol.ac.uk; 14 School of Psychology, University of Birmingham, Birmingham B15 2TT, UK; c.manning@bham.ac.uk; 15 School of Psychology, University of Sussex, Brighton BN1 9QH, UK; j.maule@sussex.ac.uk; 16 Pacific Northwest National Laboratory, Portland, OR 97204, USA; naomimiller135@gmail.com; 17 Opticalm Visual Stress Clinic, Kanata South Business Park, 150 Terence Matthews Crescent, Suite A2 2nd Floor, Ottawa, ON K2M 1X4, Canada; kmonet@opticalm.ca; 18 School of Social Sciences, Nottingham Trent University, Nottingham NG1 5LT, UK; 19 Bridging Research in AI and Neuroscience, Computer Vision Center, 08193 Bellaterra, Spain; penacchio@cvc.uab.cat; 20 Computer Science Department, Universitat Autònoma de Barcelona, 08193 Bellaterra, Spain; 21 Department of Translational Neuroscience and Stroke, University College London, London WC1N 3BG, UK; g.plant@ucl.ac.uk; 22 School of Psychology, Cardiff University, Cardiff CF10 3AT, UK; powellg7@cardiff.ac.uk (G.P.); priceaj6@cardiff.ac.uk (A.P.); 23 Department of Psychology, Aston University, Birmingham B4 7ET, UK; a.schofield@aston.ac.uk; 24 Department of Telecommunications, Faculty of Electrical Engineering and Computer Science, VSB—Technical University of Ostrava, 17. Listopadu 15/2172, 708 33 Ostrava, Czech Republic; miroslav.slouka@vsb.cz; 25 indie Technologies Switzerland AG (EXALOS), Wagistrasse 24, 8046 Zurich, Switzerland; 26 Cardiff University Brain Research Imaging Centre (CUBRIC), School of Psychology, Cardiff University, Cardiff CF24 4HQ, UK; sumnerp@cardiff.ac.uk; 27 Department of Architecture, University of Cambridge, Cambridge CB2 1PX, UK; crv29@cam.ac.uk; 28 School of Sport, Exercise and Health Sciences, Loughborough University, Loughborough LE11 3TU, UK; t.wilcockson@lboro.ac.uk; 29 Graduate School of Humanities and Social Sciences, Hiroshima University, Higashihiroshima 739-8521, Japan; syoshimo@hiroshima-u.ac.jp

**Keywords:** visual discomfort, visual stress, image statistics, flicker, phantom array, lighting, efficient encoding, precision tints, cortical excitation, sensory sensitivity

## Abstract

Visual discomfort or visual stress is an uncomfortable subjective experience that occurs in response to specific visual stimuli. It affects a large proportion of the population to various degrees, disproportionately impacting those with heightened sensory sensitivities, particularly neurodivergent individuals. We argue that this might stem from a mismatch between the statistical properties of visual stimuli in human-made environments and those in natural environments that the visual system can process efficiently. We discuss the inefficiency with which images with certain spatial, chromatic and temporal characteristics are processed by the visual system and propose a cerebral mechanism to account for the discomfort they induce. The mechanism offers a potential explanation for the large individual differences in susceptibility to discomfort. We highlight two avenues for intervention: (1) environmental modifications aimed at reducing the prevalence of visually stressing stimuli in urban settings, and (2) individual-level strategies, such as personalised optical treatments. Addressing these challenges requires an interdisciplinary effort bridging neuroscience, vision science, interior and urban design and typography to create visually accessible and inclusive environments.

## 1. Defining Visual Discomfort

Visual discomfort is a common form of sensory distress [1]. It is often dismissed due to its subjective nature, but when severe, it reduces the accessibility of modern environments, exacerbates migraine and fatigue, and negatively impacts cognitive performance on tasks such as reading. The severe form of visual discomfort is usually referred to as visual stress [2]. While most people experience visual discomfort to some degree, its occurrence and intensity are heightened in many neurological conditions and in many neurodivergent populations, including those with autism, ADHD, dyslexia, and dyspraxia [3,4]. Historically, our understanding of visual discomfort and visual stress has been fragmented, since it is distributed across a broad range of disciplines including optometry, ophthalmology, education, neuroscience, neurology, psychology, psychiatry, engineering, lighting, design and architecture. Our aim in the current review is to provide a unifying theoretical understanding of visual discomfort and visual stress, and to articulate how this can be used to improve the environments that humans design and to manage discomfort in those people who are most severely affected.

The human visual system is remarkably well adapted to process visual images over a huge range of luminances, yet certain patterns, notably stripes, strong colour contrasts, flicker and glare can provoke discomfort in some individuals while leaving others largely unaffected [5,6]. Visual discomfort is a generic term that refers to “a subjective uncomfortable experience in response to a visual stimulus, which includes uncomfortable sensations in, on, and around the eye, in the body, and difficulties with vision” [7]. The *trait* of visual discomfort is measured using various rating scales, and the *state* of discomfort by challenge with a glaring striped pattern (Pattern Glare Test [8]). Alternative terms include: pattern glare, pattern-related visual stress, visual hypersensitivity, Meares–Irlen syndrome, Irlen syndrome, and visual stress [7]. “Irlen syndrome” is a term used with a proprietary system whose methods have not been fully described in the scientific literature. The term Meares–Irlen syndrome was introduced [9] both to give credit to the individuals who first described the syndrome [10,11] and to distinguish the methods of diagnosis from those used by the Irlen organisation. Visual discomfort probably overlaps with some other conditions, including asthenopia and computer vision syndrome. The symptoms often occur during task-based work such as reading and screen use, when they are usually attributed to ocular mechanisms, particularly those involving the focussing and associated alignment of the two eyes, implicating both the sensory and motor mechanisms involved. Here we outline a completely different and possibly complementary mechanism for discomfort and stress that is stimulus-driven and cortical, and we will refer to it as *visual discomfort and visual stress of cerebral origin*. An understanding of the potential mechanisms is derived from observations in clinical neurology, from experimental findings concerning the oxygenation of the visual cortex and from the relationship between the neural efficiency of visual processing and the statistical characteristics of visual scenes.

Visual stress can have a substantial impact on the daily life and functioning of those affected. It can limit social and functional capabilities and affect relationships; individuals develop a range of coping mechanisms to prevent symptoms [12,13].

For this review, we will describe how the stimuli that provoke the discomfort have been identified, and some of the image statistics that have led to the current understanding of the neural mechanisms responsible. There are implications for design, architecture, and educational materials, including the management of discomfort with precision tinted ophthalmic lenses.

## 2. Seizures and Discomfort

**Figure 1:**
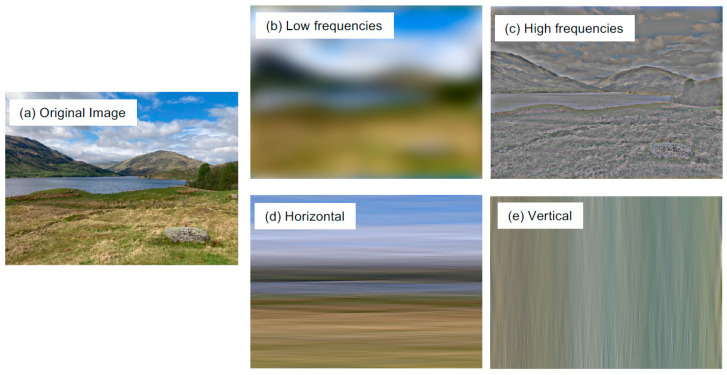
The decomposition of images via the Fourier transform. (**a**) An original image of a natural landscape. (**b**) The same scene high-pass filtered to show only its low-frequency components, resulting in a blurred version of the original. (**c**) The same scene low-pass filtered to show only the high-frequency components. The fine details are preserved, but the coarse scale differences in luminance and colour between different regions of the image are lost. The image is also shown filtered to contain only orientations close to (**d**) horizontal and (**e**) vertical. The structure of this original image is most evident in the horizontal components. The specific samples used are for illustrative purposes only and do not reflect the channel structure of the visual system.

One starting point for our understanding of visual discomfort of cerebral origin is the phenomenon of pattern-sensitive epilepsy. About 5% of patients with epilepsy are prone to seizures from flickering light and often experience visual discomfort [14]. It is not only flicker that can induce seizures, however: many patients are also at risk of seizures from repetitive patterns, particularly patterns of stripes [15]. Such flicker and patterns are rare in the natural world [16] but common in the modern urban environment [17]. The characteristics of the stripes are nevertheless very specific. The risk of seizures increases with the contrast and luminance of the stripes and with the size of the pattern, increasing linearly with the area of the visual cortex to which the stripes project (Figure 8 of reference [18]). Seizures are most likely when the stripes have an even width and spacing and when their spatial frequency is close to 3 cycles per degree subtended at the eye (Figure 1 of reference [18]). The same patterns can also provoke attacks of migraine [19].

## 3. Discomfort in the General Population

The patterns and flicker that can provoke seizures and migraine in some individuals can evoke in others discomfort, unease and a variety of visual distortions, such as illusions of colour, shape and motion. While discomfort, distortions and illusions do not invariably co-occur, all three are induced by the very same pattern characteristics that increase the risk of seizures [5]. The patterns and flicker that have these unfortunate consequences are strong stimuli psychophysically. At low contrast, they are the stimuli that can be seen most readily [20], and at high contrast they evoke (1) a large electrophysiological response in early visual cortical areas [21,22] and (2) a large haemodynamic response, measured using functional magnetic resonance imaging (fMRI) [23] or near infra-red spectroscopy (NIRS) [24]. Taken together, these results suggest that the more uncomfortable patterns create stronger neural activity, and thus impose high metabolic demands.

Recent work with high-contrast flickering sinusoidal gratings identified a degree of dissociation between visual sensitivity and discomfort. Hibbard et al. [25] showed that, for large modulation depths, variation in temporal frequencies between 1 and 16 Hz had a substantially stronger influence on discomfort than variation in spatial frequency between 0.5 and 8 cycles per degree, producing tuning functions that diverged from predictions based on the spatio-temporal contrast sensitivity function. Evidently visual sensitivity at low contrasts may not be a reliable predictor of discomfort for high-contrast patterns. Flicker is generally more aversive than spatial patterns [2], and is particularly aversive at frequencies close to 20 Hz, even at modulation depths of a few percent [26].

## 4. Efficient Coding and the Fourier Transform

The uncomfortable patterns and flicker have their effect because they trigger a large neural response (Chapter 4 of reference [2]). One current view is that the response is large because the characteristics of the patterns differ from those of images from nature. It is typically assumed that the visual system is adapted to process natural images efficiently over evolutionary, developmental and behavioural timescales [27,28,29,30,31]. Efficient encoding maximises the useful information provided, and minimises the metabolic costs involved [29,32]. Efficient encoding is possible because images of natural environments, such as landscapes, forests, rivers, and coastlines, tend to share certain temporal and spatial constraints in regard to contrast of both luminance and colour, such as characteristic Fourier amplitude spectra and wavelength spectra [16,31,32,33].

The basic properties of natural images are captured by the Fourier transform (Figure 1), a mathematical function that decomposes an image into its spatial frequency components at various phases, revealing how patterns at different spatial scales and orientations contribute to the overall structure. These statistics have allowed us to quantify how visual encoding is optimised for natural images. Conversely, they also explain how deviations from these statistics contribute to visual discomfort. The image intensity typically varies across space, and high spatial frequencies correspond to fine details such as textures, while low spatial frequencies represent smooth, broad areas like shadows, gradients, and differences in the reflectance of objects. One of the fundamental characteristics of an image is the relationship between luminance contrast and spatial frequency. In scenes from nature, contrast typically decreases as spatial frequency increases. On logarithmic scales, the decrease has a slope close to −1 (i.e., 1/f. [33,34]). As the separation between positions in space increases, the average contrast between them also increases. This reflects the fact that the luminance differences that occur within an object tend to be smaller than those between the average luminances of distinct objects [35].

Many of the fundamental characteristics of visual encoding can be understood as evolutionary adaptations that optimise processing for images with these properties. For example, the contrast sensitivity function, which describes our ability to detect low-contrast stimuli of different spatial frequencies, shows a peak in sensitivity to midrange spatial frequencies around 2–5 cycles per degree [20]. The shape of the contrast sensitivity function is optimal for images with a natural 1/f amplitude spectrum, balancing the efficient transmission of useful information with the need to minimise noise [36,37,38,39,40].

At a neuronal level, efficient processing is achieved by sparse coding, in which a small number of neurons are activated by a given stimulus [41,42,43,44]. This sparsity is achieved through decorrelation of neural responses to increase their statistical independence, emphasising the salient features of the stimulus that are most important for behaviour [45]. The ability to create a sparse and efficient code critically depends on an accurate model that anticipates the statistical properties of (natural) stimuli. Stimuli that accord well with the anticipated statistical properties can be processed efficiently. This creates a vulnerability such that, when stimuli deviate from those in nature, as they often do in the contemporary environment, the encoding mechanisms may become inefficient, creating an “out-of-gamut” response [26].

## 5. Discomfort and Spatial Frequency

**Figure 2:**
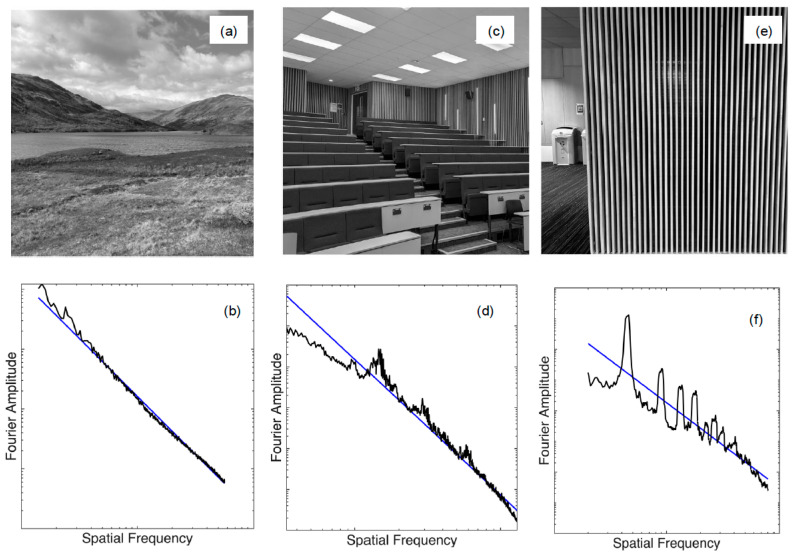
(**a**) A typical natural vista with (**b**) a characteristic 1/f pattern, the orientation-averaged log Fourier amplitude decreasing in a regular way with increasing log spatial frequency. (**c**) An indoor scene with a highly repetitive structure which (**d**) shows deviations from this regular 1/f pattern. (**e**) A close-up image of sound baffling again shows clear deviations from a regular 1/f amplitude spectrum (**f**). Amplitude spectra in all cases were plotted using the SpecSlope.m function [52].

Stimuli in which the slope of the Fourier amplitude is steeper or shallower than that encountered in natural real-world scenes tend to be judged as more uncomfortable [46]. Indeed, nearly half the variance in judgements of discomfort from photographs, paintings, or images of random noise can be predicted from their departure from a 1/f Fourier amplitude spectrum [47]. The most uncomfortable images tend to include high contrast at midrange spatial frequencies between 3 and 9 cycles per degree of visual angle [48,49,50] (see Figure 2). This is rare in natural images and coincides with the spatial frequency tuning of seizure probability [51]. As noted above, these are the spatial frequencies to which the visual system is generally most sensitive, and to which it responds most strongly.

## 6. Discomfort and Orientation

Another key characteristic of large-vista natural images is the way that amplitude varies with orientation, being maximal for cardinal (horizontal and vertical) orientations and minimal for oblique [53], and having low edge-orientated entropy. Human sensitivity to oriented structure in images matches these statistics, being highest for oblique orientations, reflecting an increase in contrast gain to compensate for the lower signal strength compared with horizontal and vertical orientations [54,55]. Burke and Longo [50] have shown there is greater discomfort for gratings with oblique as opposed to cardinal orientations. Further, the information conveyed by the orientation of edges (edge orientation entropy) in visual noise and everyday scenes is associated with judgments of unpleasantness [56] and with the brevity of “looking time” in both adults and infants [57].

## 7. Complex Images and Disgust

It is not only the scale and orientation of features that require description, but also how these components are arranged. The Fourier phase spectrum describes how the components of the amplitude spectrum line up, and determines the presence of recognisable shapes, such as edges, outlines, and the position of objects. Phase is therefore critical for determining the overall structure of the image, and the presence and location of local features. This spatial structure contributes to visual discomfort when the semantic features of the image play a critical role.

The influence of the amplitude and phase spectra on discomfort can be disentangled by creating hybrid images formed from the amplitude spectrum of one image and the phase spectrum of another. In some cases [51], visual discomfort is driven primarily by the properties of the amplitude spectrum. Here, discomfort is likely to result from the increased energy at midrange spatial frequencies. There are cases, however, in which discomfort is mixed with disgust, as in *trypophobia*, an aversion to clusters of holes. Here the meaning of the stimulus plays a role, as when the holes appear on skin, suggesting disease. Trypophobia can be disrupted by scrambling the phase of images while keeping their amplitude spectrum unchanged [58,59]. In this case, while the information responsible is found in the low and midrange spatial frequencies [60,61,62], it is the local image structure defining the cluster of holes that has an important role in determining the aversive nature of the pattern.

## 8. Colour and Differences in Colour

Discomfort can result both from the overall colour of a scene and from the difference in colour between the component surfaces. At photopic levels of illumination, flicker and patterns that contain long-wavelength red light are typically more uncomfortable to view [24,46,63,64,65]. Deep red flicker is also more likely to provoke seizures [66]. The evidence that red is particularly noxious may relate to its relative rarity in natural scenes [65,67,68,69]. The visual gamma response, which may reflect cortical excitation, is particularly strong for long-wavelength (red) stimulation, relative to other wavelengths, although still weaker than that for achromatic gratings [70].

There is a linear relationship whereby discomfort increases with the difference in colour (technically UCS chromaticity) between neighbouring surfaces, as occurs both over time and space, for instance in chromatic flicker [65,71], in static noise and Mondrian patterns [46], and in static grating patterns [24]. The effect of colour difference occurs regardless of how colour space is sampled. It occurs with or without luminance differences between the component colours, and with respect to the components of complex images such as contemporary art and domestic scenes [17,63,64,72,73,74]. Large colour differences are relatively uncommon in the natural environment [46,73] and they are conspicuous. The conspicuity of fruit in trees may have been responsible for the evolution of the long-wavelength retinal photoreceptors [75] and for a consequent increase in the associated neural signal. Perhaps the strength of the neural signal associated with conspicuous colour contrast is responsible for the discomfort. Conspicuous aposematic patterns that warn off potential predators often include strongly contrasting colours [76].

## 9. Flicker and Eye Movements

At frequencies around 16 Hz, flicker can be seen when the temporal variation in light level (modulation) is very slight. At higher modulation depths, flicker at these frequencies can induce anomalous visual effects, discomfort and seizures [2]. Discomfort from flicker is maximal at around 19 Hz for luminance-defined stimuli, 10 Hz for red–green stimuli and 9 Hz for yellow–blue stimuli, aligning with peak cortical responses [26]. Discomfort is also higher when the temporal amplitude spectrum departs from a 1/f profile, especially when this departure involves an increase in amplitude at around 15 Hz [77,78].

At frequencies above 90 Hz, temporal light modulation (TLM) is not visible as flicker, but can nevertheless create a spatial pattern as the image of illuminated contours is swept across the retina during a saccade. The spatial pattern is known as the phantom array, and individuals with migraine find it particularly aversive [79]. TLM also interferes with the perception of moving objects (the stroboscopic effect [80]). During a saccade, visual sensitivity is temporarily reduced by masking from pre- and post-saccadic retinal images, and also by active, centrally driven processes that modulate neural activity in visual areas [81]. The phantom array can be seen despite such saccadic suppression, particularly when it differs from the visual scene upon which it is superimposed, for example at night. Many car lights are modulated at 200 Hz which can make the phantom array annoyingly visible [82]. The phantom array can also interfere with reading [83], possibly by interacting with the spatially periodic patterning of text. This may be one reason why TLM interferes with eye movements across text [84].

There are large individual differences in the ability to see the phantom array, but those individuals who can see it tend to report visual discomfort in everyday life [6]. The differences in ability to see the phantom array are likely to result from a combination of the two-fold differences in saccade velocity within the normal population [85] and the four-fold differences in contrast sensitivity [86] (see [87]). The discomfort from TLM is consistent with the distribution of neural activity recorded by Lindén et al. [88] in response to high-frequency TLM.

## 10. Glare

*Disability glare* is caused by intraocular light scatter, which creates a veiling luminance that reduces contrast and obscures detail. In contrast, *discomfort glare* is the subjective experience of annoyance, strain, or pain produced by bright light sources or large luminance differences, even when visibility is preserved [89]. Given that the BOLD response in the visual cortex is larger in observers who experience discomfort glare [90], the mechanisms may be shared with the visual discomfort and visual stress of cerebral origin.

An important source of both forms of glare is from car headlights [91], dependent on the angle between the line of sight and the direction of the source [92], and its colour temperature, with bluer headlamps producing more discomfort glare [93]. The recent generation of car headlamps uses temporal light modulation at frequencies above the critical fusion frequency, and has been associated with complaints [94]. Flicker at these high frequencies has recently been shown to increase the BOLD response in the visual cortex [88].

## 11. Ocular Motor Factors and Binocular Coordination

Ocular motor processes play an essential role in seeing. Accurate lens accommodation is required for an image to be focussed on the retina, and binocular (con)vergence is necessary to ensure that the images from the two eyes are aligned at the viewing distance, and that the disparities are within Panum’s fusional area where stereopsis occurs. Anomalies in accommodation or binocular vergence can lead to a class of visual discomfort with a primarily motoric origin, which may be distinct from the cortical origin described here [95,96]. There is however an interaction between the mechanisms underlying discomfort from binocular anomalies and the visual discomfort and visual stress of cerebral origin. Binocular coordination is a computationally complex task, likely to place a high processing and metabolic demand on cortical and subcortical areas. Binocular coordination is not confined to the early visual cortex alone. While disparity processing begins in the visual cortex, the transformation of binocular signals into ocular motor commands involves a distributed network including the posterior parietal cortex, frontal eye fields, midbrain structures such as the superior colliculus, and brainstem ocular motor nuclei [97]. Visual discomfort may occur when challenging computational demands are placed in this distributed network. For example, the challenging task of processing images from 3-D displays that create an accommodation–convergence conflict can cause symptoms typically associated with binocular vision anomalies and with poor binocular function such as that which occurs in strabismus (squint or heterotropia [98,99]). Even when no inherent conflict is present, Wilkins and Evans [96] hypothesised that the perceptual instability associated with visual discomfort can in some cases interfere with the feedback required for the sensory fusion of the monocular images, which in turn can cause binocular instability. In other words, there may be a bidirectional causal relationship whereby impaired binocular coordination may contribute to visual discomfort of cerebral origin and visual discomfort of cerebral origin may contribute to ocular motor anomalies. This concept is supported by the discomfort generated in individuals with normal binocular function when viewing binocular image pairs that are imperfect (i.e., have stereo imperfections [100]).

## 12. Computational Models of Discomfort

The above work is complemented by neurocomputational models that seek to identify the mechanism through which cortical discomfort occurs. Hibbard and O’Hare [27] developed a neural encoding model in which they evaluated the expected population response of the primary visual cortex to natural images, and striped repetitive images known to be uncomfortable. They used this model to show that uncomfortable images are associated with both an increase in the overall response and a reduction in its sparseness. This approach was developed further by Penacchio et al. [28] to incorporate excitatory and inhibitory populations and biologically realistic lateral connections. They showed that the overall response, the sparseness, and the degree to which activity was evenly distributed across modelled hypercolumns were all good predictors of discomfort, especially when taken in combination. A significant advantage of this dynamic model is that it can also account for the effects of individual differences in cortical dynamics. Reducing the strength of inhibitory interconnections increased the predicted susceptibility to discomfort. Such reduced inhibition, which might result from a reduction in the inhibitory neurotransmitter GABA, has been proposed as a key mechanism in altered sensory processing in autism and migraine [101], which have both been linked to increased sensory discomfort.

## 13. Electrophysiology and the Haemodynamic Response

Stimuli that cause discomfort due to deviations from the statistics expected of natural environments tend to evoke large metabolic and electrophysiological responses in the visual cortex. Le et al. [102] used near infra-red spectroscopy (NIRS) to show a relationship between ratings of discomfort from visual images and the magnitude of the haemodynamic response to those images. Dogan et al. [103], Gentile and Aguirre [26], Haigh et al. [63], Jefferis et al. [104], O’Hare et al. [105,106], O’Hare and Hibbard [107], and Tempesta et al. [108] all used electroencephalography to investigate the relationship between the magnitude of the evoked potential and discomfort from the stimulus. Most studies demonstrate a link between cortical hyperexcitation and the stimulus properties that cause discomfort, consistent with the encoding framework outlined above. This link may even be responsible for the discomfort from glare, given that glaring light increases the cortical BOLD response in susceptible people [90]. The studies that showed the opposite tendency did not measure the total haemodynamic response but used local regions of interest [109]. A recent study assessing the levels of inhibitory neurotransmitter GABA and excitatory neurotransmitter glutamate in the visual cortex, which together modulate the overall level of activity, found mixed evidence for their association with visual discomfort as measured by the Pattern Glare Test [110], suggesting that the relationship to excitation–inhibition balance remains unsettled.

## 14. Individual Differences

While the stimulus properties that provoke discomfort are well described, it is less well understood why some people experience more discomfort than others. There are large individual differences in susceptibility to uncomfortable patterns. Some observers immediately avert their gaze whereas others experience no discomfort. Discomfort from striped patterns is greater in the young [3], in individuals with frequent headache [5], in those with migraine, particularly if they experience photophobia [111], and in individuals who are neurodivergent [3]. Tempesta et al. [108], Jefferis et al. [104] and Dogan et al. [103] combined EEG measures with questionnaire measures of visual discomfort and headache and found that headache affects the shape of the evoked response, and both habituation and sensitisation occurred in response to repeated pattern glare stimuli.

Many questionnaires have been designed to measure the individual differences in susceptibility, for example: the Visual Discomfort Scale [112]; the Cortical Hyperexcitability Index [113]; the Leiden Visual Sensitivity Scale [114]; the Utah Photophobia Symptom Impact Scale [115]; the Visual Ergonomics Risk Assessment Method [116]; the Visual Discomfort Questionnaire [7]; the Ulster Visual Stress questionnaire [117]; and the Cardiff Hypersensitivity Scale [118]. The measurement of visual discomfort also forms part of many cross-modal sensory scales that are used in clinical research, most often in autism. Common examples include the Adult Sensory Profile [119], the Glasgow Sensory Questionnaire [120] and the Multi-Modal Evaluation of Sensory Sensitivity [121]. When the questionnaires from Conlon [112], Braithwaite [113] and Perenboom [114] above were administered to 30 non-clinical participants, the total scores showed intercorrelations exceeding 0.7, suggesting variance in common across these measures [50].

## 15. Neurodiversity, Neurological Conditions and Sensory Sensitivity

Heightened sensory sensitivity (of which visual discomfort is a component) is prevalent across a broad range of subgroups linked to neurodiversity (autism, ADHD, dyslexia and dyspraxia), mental health (anxiety, depression, PTSD, eating disorders) and neurological or other conditions (epilepsy, migraine, fibromyalgia, traumatic brain injury, persistent postural perceptual dizziness, Tourette’s syndrome). A relationship between heightened sensory sensitivity and autism, and autistic traits more generally, is well established [122]. The perceptual distortion in striped patterns (in the Pattern Glare Test) has been found to be higher in diagnosed autism [122] and in depression [123]. Indeed, pattern glare predicts later symptoms of anxiety and depression [124].

Recent work has sought to clarify whether these experiences are similar across diagnostic groups. Price et al. [118] investigated this possibility using the Cardiff Hypersensitivity Scale, with bifactor modelling to define four subtypes (factors) of visual sensitivity, including sensitivity to (1) *Patterns* (e.g., high-contrast stripes), (2) *Brightness* (e.g., glare, sunlight), (3) *Strobing/Motion* (e.g., flickering lights, on-screen motion), and (4) *Intense Visual Environments* (e.g., supermarkets). The prevalence of these subtypes was investigated in 11 clinical diagnoses and areas of neurodiversity (autism, ADHD, dyslexia, fibromyalgia, migraine, synaesthesia and various mental health conditions), and a remarkably similar pattern of aversion to each type of visual input was shown across diverse diagnostic areas. The nature of visual discomfort therefore does not appear to differ instructively across conditions, suggesting a transdiagnostic pattern with variations only in magnitude. This may suggest a common underlying vulnerability to uncomfortable visual experiences across diverse symptomologies.

Susceptibility to over-excitation of the cortex, as revealed in the studies described above, is likely to explain some of the individual differences, but it may be wise to also consider connectivity with wider brain networks. For example, greater activation of the amygdala and other limbic regions has been reported for aversive visual and auditory stimuli in autism, and sensory sensitivity is correlated with individual differences in activation in both autism and neurotypical controls [125]. Green et al. [126] found increased connectivity between the thalamus and amygdala correlated with sensory hypersensitivity in autism. Connectivity differences have also been investigated after observation of uncomfortable images of stripes, and they outlast the stimulus presentation [127]. In individuals with migraine there are differences in the connectivity of the visual pathways between one headache and the next [128]. In migraine with aura, differences in visually evoked potentials [129,130], attenuated alpha oscillation [131] and increased background activity [132] have also been reported.

## 16. Design

Understanding why some visual stimuli are uncomfortable provides us with an opportunity to design buildings, text, lighting and screens to minimise visual discomfort. It is important to note that the design decisions required to reduce discomfort are often cost-neutral, and if made at an early stage can avoid the retro-fitting or redesign required to address the discomfort.

Architectural design has traditionally been evaluated according to the extent that it provides strength and stability, meets its functional requirements, and provides a positive aesthetic experience [133,134]. Vaughan [135] has argued that, for the past century, the needs of physical safety, economy and utility have been prioritised over other considerations, such as sensory effects. The extent to which urban design is comfortable and resembles that in nature can be measured by the goodness of fit to a 1/f Fourier amplitude spectrum. Wilkins et al. [17] used this metric in an analysis of images of apartment buildings sourced from Google and showed that over the last century, design has become progressively less like that in nature. The progression is particularly evident in successive epochs of Korean architecture [136]. It is important in biophilic design to recognise and reduce this adverse progression [137,138].

Le et al. [102] used the same metric in an analysis of images of building frontages and showed that images with close adherence to 1/f not only were rated as more comfortable to look at, but also elicited a smaller cortical haemodynamic response, suggesting that they were more efficiently processed by the brain [109]. They also showed that pictures of buildings and open spaces were rated similarly to ratings of the buildings and spaces themselves.

Repetitive patterns occur not only on the outside of buildings but also within them [17]. The use of repetitive patterns in grills, grids, blinds, mats, radiators and acoustic panelling is commonplace. When repetitive patterns cannot be removed, their visual effects can be greatly reduced by decreasing the contrast between the elements. This is not always possible, however. Acoustic panelling sometimes uses repetitive patterns of holes or grooves with a scale dictated by the wavelength of sound, and such panelling has resulted in visual stress in lecture theatres (Hosking, personal communication). Black panelling would reduce the contrast of the holes but would reflect insufficient light. It may therefore be necessary to use a different physical design.

Recent advances in visual discomfort assessment software [139] provide designers and planners with quantitative tools to evaluate façades, interiors, and urban surfaces for spectral properties known to elicit visual discomfort. These tools offer a means of using visual ergonomics as a design parameter rather than a post hoc aesthetic judgement. Integrating such evidence-based assessment into design practice therefore represents a critical step in aligning architectural decision-making with health promotion, ensuring that environments support rather than compromise perceptual comfort and wellbeing.

There is an informative parallel for visual discomfort with a disorder known as “visual vertigo”. Sufferers complain of discomfort when moving through visually busy environments—supermarkets being a typical example. Some are no longer able to drive. There is minimal evidence of vestibular dysfunction in most patients and a programme of repeated exposure to such visual stimulation can improve symptoms. A very subtle mismatch between vestibular and ocular motor function results in disabling discomfort when exposed to visual surroundings similar to those discussed above [140].

## 17. Text and Reading

Text is an unnatural, achromatic, repetitive pattern. The successive horizontal lines of printed text when spatially averaged have a Michelson contrast that varies between 12% and about 22% [141], which is therefore sufficient to induce distortions/illusions and discomfort. The greater the spacing between the lines relative to the height of the central body of the letters, the lower the Fourier power in the striped pattern. The opportunities for personalisation of displays in electronic books provides a novel form of data on how people adjust text to minimise discomfort. When readers adjust iBooks to improve the readability of text, their adjustments reduce the departure of the Fourier amplitude spectrum of the resulting images from 1/f [142]. The Fourier spectrum provides a better prediction than individual typographic measures such as x-height, line spacing and the use of serifs.

The vertical strokes of letters in certain words, such as “mini” also comprise a repetitive pattern and this has been shown to interfere with the realignment of the eyes following a saccade [143]. The repetitive nature of letter strokes can be measured using the first peak in the horizontal auto-correlation of words. Passages in which the words have high auto-correlation are read more slowly [144] possibly because the pattern slows the realignment of the eyes after a saccade [143]. The auto-correlation is generally greater for serif fonts [142], and in some fonts designed for children [145]. Text in children’s reading schemes gets too small too early in life, compromising reading speed [146]. In consequence of the above, some individuals see perceptual distortions in text, including apparent movement of the words and intrusive shapes and colours [147]. The music stave is also a striped pattern. Sometimes the lines are insufficiently fine and impair musical performance when sight-reading [148].

## 18. Lighting

Perhaps the most important difference between natural environments and those that are made by humans concerns the way in which they are lit. Electric lighting varies continuously and invisibly twice with each cycle of the alternating current. In the days when electric lighting was incandescent, the hot filament retained most of the light from one cycle of the electricity supply to the next, reducing the temporal light modulation (TLM) well below 20% [149]. With the introduction of gas discharge lighting in the 1950s, the modulation was greater, but it was more than forty years before it was shown that the 100 Hz modulation from fluorescent lighting causes headaches [150]. We now know that the effects of 100 Hz temporal light modulation can be detected in the visual cortex of the brain by BOLD activation [88]. In individuals with migraine, the activation involves the periaqueductal grey, an area often associated with pain [151]. This indicates that some individuals are more sensitive than others and provides a mechanism for the induction of pain. A change to electronic control circuitry reduced the 100 Hz temporal modulation from fluorescent lamps but the benefit has been short-lived owing to the introduction of Light-Emitting Diode (LED) lighting. Early LED lamps in the USA were driven by the 60 Hz AC supply with no smoothing. Most drivers now use various techniques to provide DC, albeit with various degrees of ripple [152]. In early LED installations the modulation at 100 Hz and 120 Hz was poorly controlled: the variation in light level over time was often complex involving a wide range of frequency components [153]. More recently improvements have been made in the design of the control circuitry. Lamps with little temporal modulation are now available, although there remains a legacy of poor lighting.

Computer screens and LED lamps are often turned on and off at high frequency and the proportion of each cycle during which the light is turned on can then be varied to control the brightness (a technique known as pulse-width modulation, PWM). The phantom array from rapid flicker has been shown to impair reading [83] which would suggest that PWM should not be used at frequencies less than 20 kHz, which is beyond the upper frequency limit of perception of the phantom array (obtained both theoretically and empirically [87]). PWM can, in principle, be generated at frequencies in the MHz range, but the emission characteristics of semiconductor light sources are then affected [154,155]. In illumination-class LED drivers, PWM in the range 10–100 kHz becomes challenging because of the minimum achievable on/off times [156], the finite current rise/fall dynamics at low dimming levels [157], and the switching-loss and electromagnetic interference [158,159]. An ideal option is to avoid pulses entirely and control intensity continuously by reducing the analogue current, although this can alter output characteristics, especially colour [160]. A widely used compromise is hybrid dimming in which brightness is reduced continuously by analogue control down to the point where chromatic stability becomes limiting, and PWM is then used only for deep dimming to extend the usable range without demanding extremely small regulated currents [161,162], as in automotive systems that require multiple discrete intensity levels [163].

Although the spectral power distribution of LED lighting can be varied at will using combinations of different sources, most general-purpose LED lamps are designed to produce white light rather than strongly saturated colours. The spectral power has a sharp peak at about 450 nm and a compensatory shallow peak at longer wavelengths, depending on the desired colour. Some observers prefer a cool white light (CCT~4000 K) and others a warm white (CCT~3000 K), with the preference dependent on location, time of day, and the desired atmosphere, although most people prefer chromaticities that lie on or close to the daylight locus [164]. As will now be shown, individuals who experience migraine with aura differ in their preferred choice of comfortable chromaticity.

## 19. Management of Visual Discomfort/Visual Stress of Cerebral Origin

Huang et al. [165] demonstrated an abnormally large haemodynamic response to uncomfortable grating patterns in patients with migraine. The response was normalised when the patients wore glasses tinted a colour that they had individually selected as comfortable. Their selection was made using the Intuitive Colorimeter [166], an instrument that illuminates text with coloured light and permits the user to vary the hue and saturation independently at constant luminance. Glasses with a colour that differed by 0.06 in CIE UCS chromaticity were not effective at reducing the haemodynamic response. This suggests that a limited range of alternative colours is not generally sufficient to reduce the haemodynamic response and associated discomfort. Individuals who read more quickly with coloured filters do so only when the filters have a limited gamut of CIE 1976 UCS chromaticities [167]. The dependence of discomfort on UCS chromaticity has been modelled as a bivariate normal distribution with a standard deviation of 0.02 [168].

Although healthy individuals select light with chromaticities close to the daylight locus as comfortable, individuals who experience migraine with aura have repeatedly been shown to select as comfortable chromaticities distant from the locus [169]. It has been proposed that the colour reduces the discomfort because the visual stimulation then avoids visual areas of the brain that are hyperexcitable. The hypothesis has yet to be tested. Nevertheless, the observation suggests a mechanism for management of discomfort. Indeed, glasses with the appropriate chromaticity improve not only visual comfort, but also the speed of visual search in patients with migraine aura [170].

Similar mechanisms may be responsible for the improvement in reading speed that often occurs in certain children when they use coloured overlays to cover the text they are reading. The use of coloured filters has sometimes been publicised as a treatment for dyslexia, but only a minority of individuals with dyslexia experience visual discomfort [171], and this meant that the benefits of coloured filters were dismissed [172] despite their widespread usage [173]. The overlay had a colour individually chosen from a set of at least nine different colours (a smaller set of colours was insufficient [174]). Reading speed was measured using common, simple words printed in a closely spaced paragraph that the children read aloud. Those individuals who reported improved clarity of text with an overlay usually read more quickly with the overlay on this test both before [175] and after [176] long-term use of the overlay. Overlays can also improve reading speed when conventional un-crowded prose is read. The improvement may then be measurable only after a lengthy period of reading, sufficient to reduce the effects of the variability that is due in part to comprehension [177]. Both overlays and precision tinted lenses have also been shown to improve reading and social cognition in children with autism [178,179]. The several possible neural mechanisms for the effects of coloured light have been considered, but remain uncertain [180].

## 20. Conclusions

**Figure 3:**
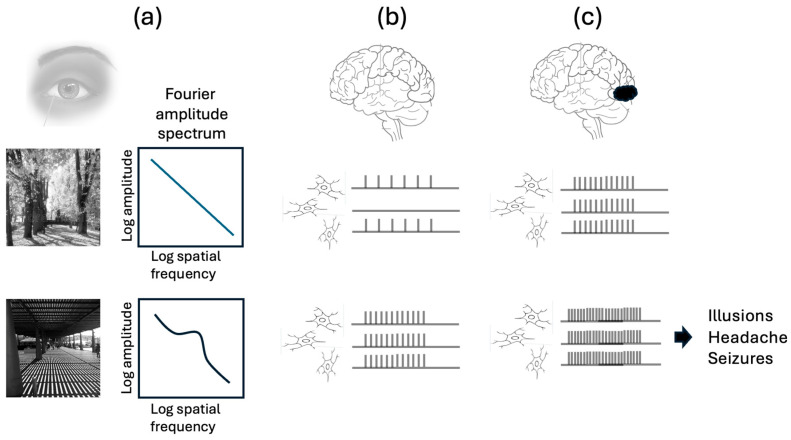
A theoretical framework for visual discomfort of cerebral origin. (**a**) The visual system is evolutionarily adapted to process natural images which possess specific statistical properties, such as a 1/f Fourier amplitude spectrum (**upper row**). Uncomfortable unnatural stimuli (e.g., urban stripes, flicker) deviate from these statistics (**lower row**). (**b**) Natural stimuli allow for sparse, efficient cortical coding with low metabolic cost. Unnatural stimuli result in inefficient encoding, causing excessive neural activity (hyperexcitability) and high metabolic (oxygen) demand in the visual cortex. (**c**) When this demand exceeds a homeostatic threshold, discomfort, distortions, or seizures occur. This threshold is modulated by individual differences in cortical inhibition (e.g., in migraine, autism, and epilepsy). Interventions such as precision tints or environmental redesign aim to align the visual input closer to physiological tolerances.

Although originally referring to illusions and pain in response to stripes [5], visual discomfort and visual stress of cerebral origin has come to refer to aversion in response to visual stimuli (i.e., patterns and flicker) with specific spatial, temporal or chromatic properties distinct from those commonly found in scenes from nature. These specific stimuli differ in their Fourier spectra as regards temporal frequency, spatial frequency, orientation, phase and spectral power distribution [59,180]. We hypothesise that the discomfort is a homeostatic response to the excessive oxygen demands of the visual cortex due to inefficient encoding of the visual stimuli [27,181]. This hypothesis provides a way to integrate our knowledge and understanding of a range of related phenomena, a mechanistic account of why they occur and how they can be mitigated, and a framework for identifying unresolved research questions (Box 1). It also provides a means of investigating individual differences in visual discomfort, particularly in relation to neurological diversity. The theoretical account we have outlined here is summarised in Figure 3, which shows how the key components of visual discomfort relate.

Box 1 **Unresolved research questions and future direction.**

- If the mechanisms of visual discomfort are cerebral in origin, it is reasonable to expect effects on visual function. These effects need to be explored; for example, what is the relationship between the unpleasant sensations and the anomalous percepts and difficulties with vision?
- How can we test the links between visual discomfort and cortical measures of excitatory and inhibitory activity, such as levels of glutamate and GABA, and the visual gamma response?
- There are large individual differences in susceptibility to discomfort. Exactly what neural mechanisms are responsible?
- Visual discomfort affects lives adversely, but how can this effect be systematically quantified?
- Current visual tests of susceptibility to discomfort are subjective and poorly standardised [182]. There is evidence for a correlation between discomfort and some more objectively measurable visual functions (e.g., pattern glare and reading speed [183]) but not others (e.g., discomfort glare and driving performance [91]). How can the objective correlates best be assessed psychophysically, and how can they improve the quantification of visual discomfort?
- How can we redesign architectural features, such as acoustic panelling, so they maintain their effectiveness but do not create visual discomfort?
- What is the relationship between the visual discomfort felt by the general population, and the debilitating effects with significant personal and societal impact (visual stress) experienced by some people?

## Data Availability

No new data were created or analysed in this study. Data sharing is not applicable to this article.
